# Rate of Intraoperative Crystalloid Administration During Thoracic Surgery Is Causal in Reducing Postoperative Hospital Length of Stay

**DOI:** 10.31486/toj.22.0113

**Published:** 2023

**Authors:** Morgan T. Smith, Ashley D. Peairs, Bobby D. Nossaman

**Affiliations:** ^1^ The University of Queensland Medical School, Ochsner Clinical School, New Orleans, LA; ^2^ Department of Anesthesiology and Perioperative Medicine, Ochsner Clinic Foundation, New Orleans, LA

**Keywords:** *Colloids*, *crystalloid solutions*, *intraoperative care*, *perfusion*, *postoperative complications*, *thoracic surgery*

## Abstract

**Background:** Studies in thoracic surgery have long raised concerns that intraoperative administration of intravenous fluids exacerbates or causes postoperative complications and hence advocate fluid restriction.

**Methods:** This retrospective 3-year study investigated the role of intraoperative crystalloid administration rates on the duration of postoperative hospital length of stay (phLOS) and on the incidences of previously reported adverse events (AEs) in 222 consecutive patients following thoracic surgery.

**Results:** Higher rates of intraoperative crystalloid administration were significantly associated with shorter phLOS (*P*=0.0006) and with less variance in phLOS. Dose-response curves showed progressive decreases in the postoperative incidences of surgical, cardiovascular, pulmonary, renal, other, and long-term AEs with higher intraoperative crystalloid administration rates.

**Conclusion:** The rate of intravenous crystalloid administration during thoracic surgery was significantly associated with duration of and variance in phLOS, and dose-response curves showed progressive decreases in the incidences of AEs associated with this surgery. We cannot confirm that restrictive intraoperative crystalloid administration benefits patients undergoing thoracic surgery.

## INTRODUCTION

The major goal of intravenous (IV) fluid administration during surgery is to maintain tissue perfusion.^1-4^ Studies in thoracic surgery have long raised concerns that intraoperative administration of IV fluids exacerbates or causes postoperative pulmonary complications and therefore advocate fluid restriction.^5-9^ However, restrictive intraoperative fluid management risks impaired tissue perfusion with development of postoperative organ dysfunction and delayed hospital discharge.^10-14^

The purpose of this retrospective study was to analyze the role of intraoperative crystalloid administration on the duration of postoperative hospital length of stay (phLOS) and on the incidence of previously reported adverse events (AEs) associated with thoracic surgery.^5-9^ A secondary goal was to evaluate the role of intraoperative administration of colloids and of packed red blood cell (pRBC) transfusion on the duration of phLOS.

## METHODS

Following institutional review board approval, we extracted data from 222 consecutive thoracic surgeries from November 2012 to February 2016. We examined the role of intraoperative crystalloid administration, when expressed as dose-response curves, on the duration of phLOS and on the incidence of previously reported AEs: surgical (unplanned surgical intensive care unit [SICU] admission, reoperation, wound infection, ileus requiring total parenteral nutrition [TPN]); cardiovascular (postoperative nonsinus dysrhythmias, postoperative hypertension, postoperative hypotension requiring therapy); pulmonary (pneumonia, respiratory failure requiring orotracheal reintubation, pulmonary embolus, pneumothorax); renal (urinary tract infection, new onset renal insufficiency); other (deep venous thrombosis, postoperative delirium); and long-term (readmission in 30 days, long-term acute care [LTAC] placement, and mortality).^5-9^

We performed an initial univariate analysis of 24 previously reported predictors (demographic characteristics, comorbidities, preoperative laboratory values),^5-9,15^ followed by bivariate analyses of these predictors with the association to phLOS. Multivariable analysis with and without an instrumental variable^16-23^ screened the association of the 24 predictors, along with length of surgery^6,8,9^ and rate of intraoperative crystalloid administration to duration of phLOS, the primary outcome of interest.^5-14^ For this study, the geographic region in which the patients reside was chosen as the instrumental variable. We also assessed the presence of multicollinearity in the independent predictors with the use of variance inflation factor (VIF) calculations.^24^ Loglinear variance was used to analyze phLOS residuals across the range of intraoperative crystalloid administration rates.^25-27^ The robust statistical technique, recursive partitioning, was employed to explore the relationship of intraoperative administration of colloids on the duration of phLOS.^27-32^ Additional tests analyzed the support for causality in the role of intraoperative crystalloid administration on duration of phLOS.^21,23,33^ *P* values <0.01 were considered statistically significant for all frequentist tests.^16,17^ Sample size calculations for multivariable analysis required a minimum of 200 patients.^22,23^ The statistical program, JMP 13.2 (SAS Institute Inc.) was used for this study.

## RESULTS

### Association of Previously Reported Demographic and Clinical Characteristics and Length of Surgery on Hospital Length of Stay

The 24 baseline demographic and clinical characteristics in 222 consecutive patients who underwent thoracic surgery are presented in Table 1 as summations and with bivariate analyses of the association between these predictors to phLOS. Two independent predictors demonstrated significant statistical association with duration of phLOS: chronic obstructive pulmonary disease (COPD) and preoperative albumin.

**Table 1.** Association of Baseline Predictors on Postoperative Hospital Length of Stay (phLOS) in 222 Consecutive Patients Undergoing Thoracic Surgery

Baseline Predictor	Summation	phLOS	*P* Value
**Demographic**			
Age, years, median [IQR]	64 [54-71]	*^a^*	0.0637
Sex, male	114 (51)		
Male, median [IQR]		5.1 [3.3-8.5]	0.0238
Female, median [IQR]		4.1 [2.7-6.5]	
Body mass index, kg/m^2^, median [IQR]	28 [24-33]	*^b^*	0.8887
American Society of Anesthesiologists physical status classification score			
II	44 (19.8)	4.2 [3.2-6.0]	0.0170
III	154 (69.4)	4.2 [3.2-7.3]	
IV	24 (10.8)	7.3 [4.5-20.0]	
**Cardiac**			
Systemic hypertension	134 (60.4)		
Present, median [IQR]		4.4 [3.2-7.4]	0.4461
Absent, median [IQR]		4.2 [3.2-6.7]	
Coronary artery disease	38 (17.1)		
Present, median [IQR]		6.1 [3.5-8.5]	0.0642
Absent, median [IQR]		4.2 [3.2-7.3]	
Nonsinus dysrhythmias	19 (8.6)		
Present, median [IQR]		6.5 [3.4-13.2]	0.0580
Absent, median [IQR]		4.2 [3.2-7.2]	
Coronary artery bypass graft	4 (1.8)		
Present, median [IQR]		8.4 [6.5-12.0]	0.0574
Absent, median [IQR]		4.3 [3.2-7.3]	
Congestive heart failure	11 (5.0)		
Present, median [IQR]		7.2 [3.4-13.0]	0.1063
Absent, median [IQR]		4.3 [3.2-7.3]	
Cardiomyopathy	3 (1.4)		
Present, median [IQR]		7.3 [6.3-13.0]	0.1170
Absent, median [IQR]		4.3 [3.2-7.3]	
Peripheral vascular disease	20 (9.0)		
Present, median [IQR]		6.2 [4.0-10.0]	0.0389
Absent, median [IQR]		4.2 [3.2-7.3]	
**Pulmonary**			
Tobacco abuse	57 (25.7)		
Present, median [IQR]		4.4 [3.3-7.3]	0.7869
Absent, median [IQR]		4.3 [3.2-7.3]	
Chronic obstructive pulmonary disease	54 (24.3)		
Present, median [IQR]		6.7 [4.2-13.0]	<0.0001
Absent, median [IQR]		4.0 [3.1-6.5]	
Reactive airway disease	28 (12.6)		
Present, median [IQR]		5.2 [3.1-13.0]	0.5793
Absent, median [IQR]		4.3 [3.2-7.3]	
**Other**			
Diabetes	54 (24.3)		
Present, median [IQR]		5.8 [3.1-10.0]	0.3043
Absent, median [IQR]		4.2 [3.2-7.0]	
Liver disease	16 (7.2)		
Present, median [IQR]		4.6 [3.0-12.0]	0.8187
Absent, median [IQR]		4.3 [3.2-7.3]	
Renal insufficiency	18 (8.1)		
Present, median [IQR]		5.9 [2.4-14.0]	0.4529
Absent, median [IQR]		4.3 [3.2-7.3]	
**Preoperative laboratory, median [IQR]**			
Hemoglobin, g/dL	13 [11.5-14.1]	*^c^*	0.3868
Alkaline phosphatase, IU/L	83 [68-100]	*^d^*	0.0561
Alanine transaminase, IU/L	17 [12-28]	*^e^*	0.3770
Aspartate transferase, IU/L	21 [16-27]	*^f^*	0.0561
Bilirubin, mg/dL	0.5 [0.4-0.7]	*^g^*	0.3868
Albumin, g/dL	3.5 [3.1-3.8]	*^h^*	0.0002
Creatinine, mg/dL	0.8 [0.7-1.0]	*^i^*	0.0221

Notes: Data are presented as n (%) unless otherwise indicated. *P* values <0.01 are statistically significant. Bivariate analysis formulae for continuous variables are as follows: *^a^*phLOS = 3.1 + 0.06 × age; *^b^*phLOS = 7.0 × body mass index; *^c^*phLOS = 4.3 – 0.6 × hemoglobin; *^d^*phLOS = 5.1 + 0.02 × alkaline phosphatase; *^e^*phLOS = 6.4 + 0.02 × alanine transaminase; *^f^*phLOS = 5.9 + 0.04 × aspartate transferase; *^g^*phLOS = 6.3 + 1.1 × bilirubin; *^h^*phLOS = 17.2 – 3.0 × serum albumin; *^i^*phLOS = 3.6 + 3.4 × creatinine.

IQR, interquartile range.

The surgical approaches to the lung are shown in Table 2. The majority of surgical procedures were video-assisted thoracoscopies with associated procedures (61.7%). A pneumonectomy did not undergo analysis.

**Table 2.** Surgical Approach in 222 Patients Undergoing Thoracic Surgery

Surgical Approach	n (%)
Video-assisted thoracoscopy surgery with lung resection	95 (42.8)
with wedge lung resection	15 (6.8)
with open lung biopsy	6 (2.7)
with pleurectomy-partial	3 (1.4)
field examination	12 (5.4)
with sympathectomy	1 (0.5)
wedge	1 (0.5)
lobectomy	1 (0.5)
lung resection/sleeve lobectomy	3 (1.4)
Thoracotomy	37 (16.7)
lobectomy	21 (9.5)
with lung resection	11 (5.0)
mini nodule removal	1 (0.5)
opposite side	1 (0.5)
biopsy	1 (0.5)
extrapleural pneumonectomy	1 (0.5)
pleurectomy	1 (0.5)
Robotic-assisted laparoscopic wedge resection	6 (2.7)
lobectomy	3 (1.4)
pleural drainage	2 (0.9)
Total	222 (100.0)

The mean length of surgery of 2.4 hours, the 95% CI of 2.1-2.6 hours, and the SD of the mean of 1.5 hours were associated with duration of phLOS (F-ratio=15.3, *P*=0.0001).

### Role of Intraoperative Fluid Administration, Estimated Intraoperative Blood Loss, and Length of Surgery on Postoperative Surgical Intensive Care Unit and Hospital Length of Stay

**Figure 1.**
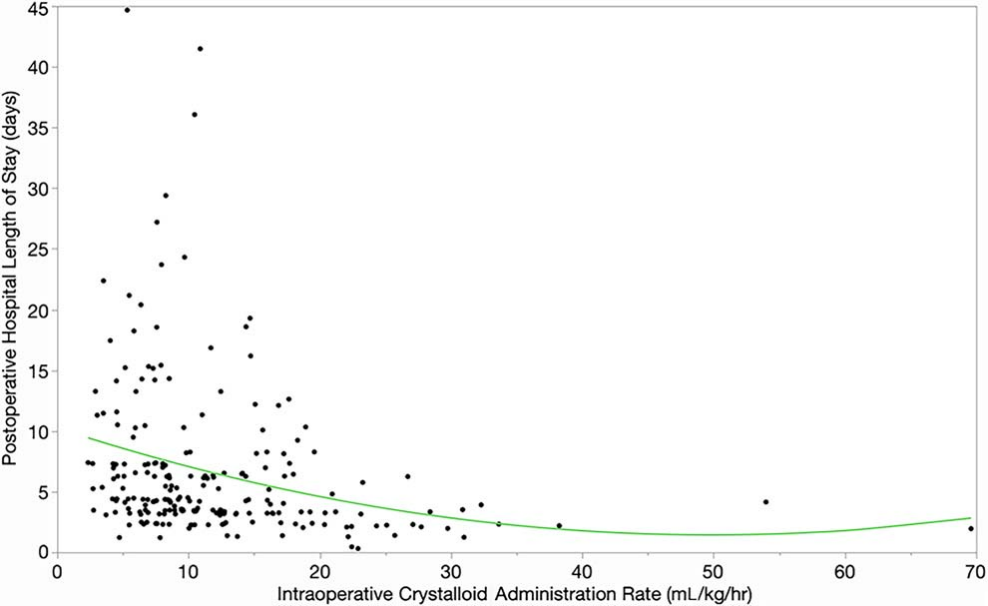
Bivariate fit of the role of intraoperative crystalloid administration (mL/kg/hr) on postoperative hospital length of stay following thoracic surgery in 222 consecutive patients. Postoperative hospital length of stay = 9.7 – 0.27 × intraoperative crystalloid administration rate + 0.004 × (intraoperative crystalloid administration rate – 12.06)^2^; F-ratio=7.6, *P*=0.0006. *P* values <0.01 are statistically significant.

The mean phLOS was 6.7 days (95% CI 6.5-7.5 days) and the SD of the mean was 6.5 days. The association between intraoperative crystalloid administration rate and duration of phLOS following thoracic surgery is shown in Figure 1. A progressive decrease in mean phLOS was observed with increasing rates (mL/kg/hr) of intraoperative crystalloid administration. This association was statistically significant (F-ratio=7.6, *P*=0.0006), with the fitted line shaped as a medical J-curve.^26,34^ Less variance was seen in the ranges of phLOS values as the rate of intraoperative crystalloid administration increased. Loglinear variance analysis of the dispersion of the phLOS residuals across the range of intraoperative crystalloid administration rates was statistically significant (estimate 46.8 mL/kg/hr; 95% CI 24.7-63.3 mL/kg/hr; standard error of the estimate 7.2 mL/kg/hr; χ^2^=42.5, *P*<0.0001).^25-27^ When intraoperative crystalloid administration rates were partitioned into mL/kg/hr tertiles, progressive decreases in the median and quantile values were observed, with comparisons statistically significant in the higher tertile groups (Table 3).

**Table 3.** Role of Intraoperative Crystalloid Administration Expressed as Partitioned Tertiles on Postoperative Hospital Length of Stay Following Thoracic Surgery^a^

		Postoperative Hospital Length of Stay, days						
Crystalloid Administration Tertile, mL/kg/hr	Count	Minimum	10%	25%	Median	75%	90%	Maximum
<11.9	134	1.2	2.4	3.5	5.3	8.6	17.1	44.7
≥11.9 to <20.4	59	1.3	2.3	3.2	4.0	7.3	12.2	19.3
≥20.4	25	0.3	0.9	2.0	2.2	3.4	5.2	6.2

^a^The patient count is 218 because 4 anesthetic records did not chart the use of intraoperative crystalloids.

Notes:

Primary partitioned cut-point 11.9 mL/kg/hr, logworth 3.7, *P*<0.0001. Secondary partitioned cut-point 20.4 mL/kg/hr, logworth 3.7, *P*<0.0001.

Nonparametric comparisons between tertile pairs utilizing the Wilcoxon method:

<11.9 mL/kg/hr tertile to ≥11.9-<20.4 mL/kg/hr tertile, Z=–2.3, 95% CI –2.1 to 0.06, *P*=0.0199;

≥20.4 mL/kg/hr tertile to ≥11.9-<20.4/ mL/kg/hr tertile, Z=–4.0, 95% CI –1.9 to –0.7, *P*<0.0001;

≥20.4 mL/kg/hr tertile to <11.9 mL/kg/hr tertile, Z=–5.7, 95% CI –4.7 to –1.4, *P*<0.0001.

*P* values <0.01 are statistically significant.

We also examined the administration of intraoperative colloids during thoracic surgery for this study. Twenty-one patients also received intraoperative colloids, either as 5% human albumin (19 patients, range 100 to 1,000 mL) or 5% hetastarch (3 patients, 500 mL); 1 patient received both colloids. In the 21 patients who received intraoperative colloids, median [interquartile range (IQR)] phLOS was 7.3 [6.2-12.4] days. In the 197 patients who only received intraoperative crystalloids, median [IQR] phLOS decreased to 4.2 [3.2-7] days (χ^2^=17, *P*<0.0001).

Recursive partitioning with 5-fold cross-validation^27-32^ identified a cut-point in the intraoperative volume of transfused pRBCs that influenced the duration of phLOS. In the 6 patients who received up to 300 mL of pRBCs during thoracic surgery, the median [IQR] phLOS averaged 4.2 [3.2-7.2] days. In contrast, in the 16 patients who intraoperatively received ≥300 mL of pRBCs, the median [IQR] phLOS increased to 7.4 [6.2-17.2] days (χ^2^=12.3, *P*=0.0005).

We examined the association of 4 indirect measures of surgical injury on the incidence of unplanned SICU admission: median [IQR] estimated blood loss (EBL) (100 [30-300] mL), mean pRBC transfusion (mean 60 mL, 95% CI 30-90 mL, SD 220 mL), length of surgery, and intraoperative crystalloid administration. These predictors were not statistically associated with unplanned SICU admission (EBL: χ^2^=4.7, *P*=0.0294; pRBC: χ^2^=0.02, *P*=0.8940; length of surgery: χ^2^=4.8, *P*=0.0283; and intraoperative crystalloid administration: χ^2^=0.2, *P*=0.6915). The administration of intraoperative colloids increased the incidence of unplanned SICU admission from 4.6% to 14.3% (χ^2^=2.6, *P*=0.1094, odds ratio 3.5, 95% CI 0.86-14).

### Association of Intraoperative Crystalloid Administration Rates on the Incidences of Postoperative Adverse Events

#### Surgical Adverse Events

**Figure 2.**
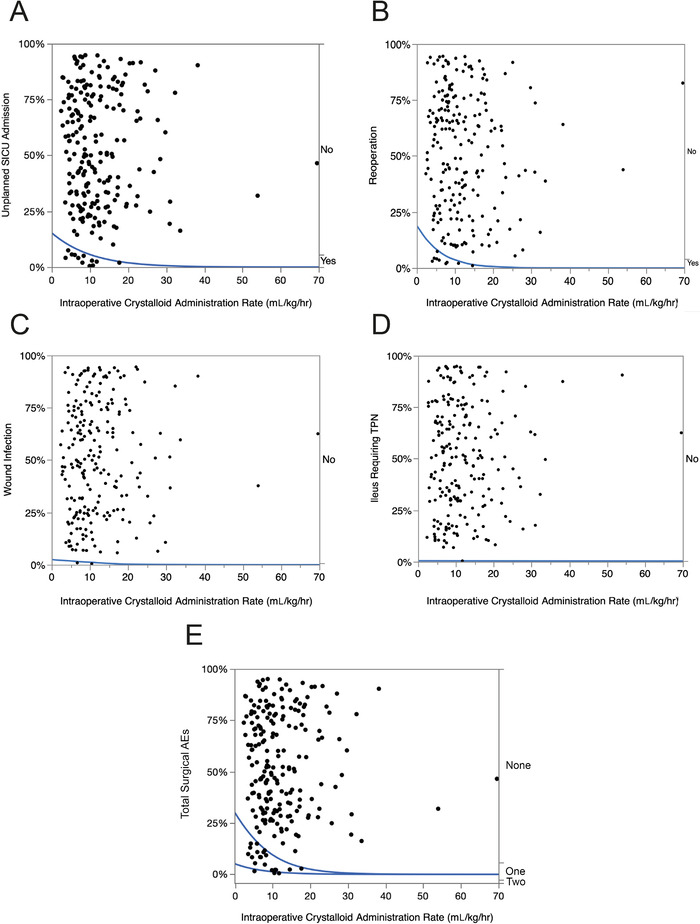
**Logistic fit of the role of intraoperative crystalloid administration (mL/kg/hr) on the incidence of common postoperative surgical adverse events (AEs) following thoracic surgery in 222 consecutive patients. The blue lines are the percent incidence fit of the dose-response relationship to surgical AEs. *P* values <0.01 are statistically significant**. **(A) Unplanned surgical intensive care unit (SICU) admission: chi-square=3.8, *P*=0.0527; (B) Reoperation: chi-square=5.8, *P*=0.0161; (C) Wound infection: chi-square=0.6, *P*=0.4528; (D) Ileus requiring total parenteral nutrition (TPN): chi-square=0.002, *P*=0.9647; (E) Total surgical AEs: chi-square=9.1, *P*=0.0026.**

Overall, the incidence of surgical AEs was 5.4% (95% CI 3.1%-9.2%). Dose-response graphs for the incidences of unplanned SICU admission, reoperation, wound infection, and ileus requiring TPN are shown in Figures 2A to 2D. When the total number of postoperative surgical AEs (unplanned SICU admission [12 patients], reoperation [9 patients], wound infection [2 patients], and ileus requiring TPN [1 patient]) was plotted against the rate of intraoperative crystalloid administration (Figure 2E), all AEs occurred at lower rates of intraoperative crystalloid administration.

#### Cardiovascular Adverse Events

**Figure 3.**
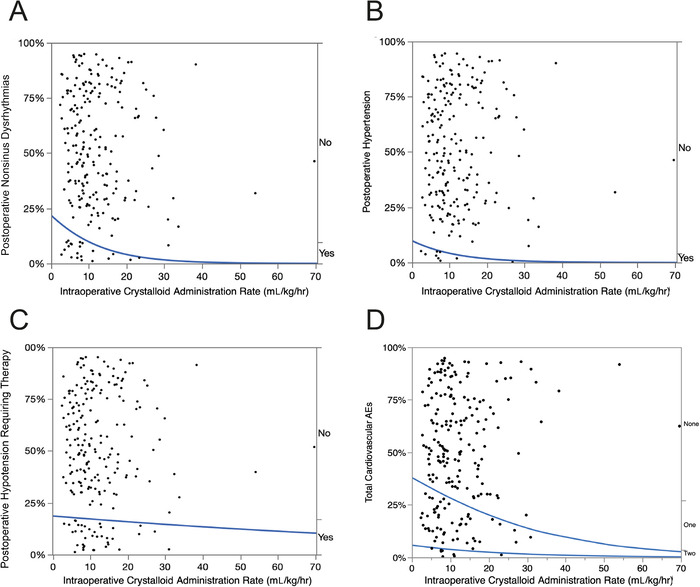
**Logistic fit of the role of intraoperative crystalloid administration (mL/kg/hr) on the incidence of common postoperative cardiovascular adverse events (AEs) following thoracic surgery in 222 consecutive patients. The blue lines are the percent incidence fit of the dose-response relationship to cardiovascular AEs. *P* values <0.01 are statistically significant**. **(A) Postoperative nonsinus dysrhythmias: chi-square=5.2, *P*=0.0229; (B) Postoperative hypertension: chi-square=2.2, *P*=0.1381; (C) Postoperative hypotension requiring therapy: chi-square=0.18, *P*=0.6721; (D) Total cardiovascular AEs: chi-square=4.0, *P*=0.0446.**

Overall, the incidence of postoperative cardiovascular AEs was 30% (CI 24.4%-36.4%). Dose-response graphs for the incidences of postoperative nonsinus dysrhythmias, postoperative hypertension, and postoperative hypotension requiring therapy are shown in Figures 3A to 3C. When the total number of cardiovascular AEs (postoperative nonsinus dysrhythmias [21 patients], postoperative hypertension [9 patients], and postoperative hypotension requiring therapy [37 patients]) was plotted against the rate of intraoperative crystalloid administration (Figure 3D), these AEs, except for postoperative hypotension requiring therapy, occurred at lower rates of intraoperative crystalloid administration.

#### Pulmonary Adverse Events

**Figure 4.**
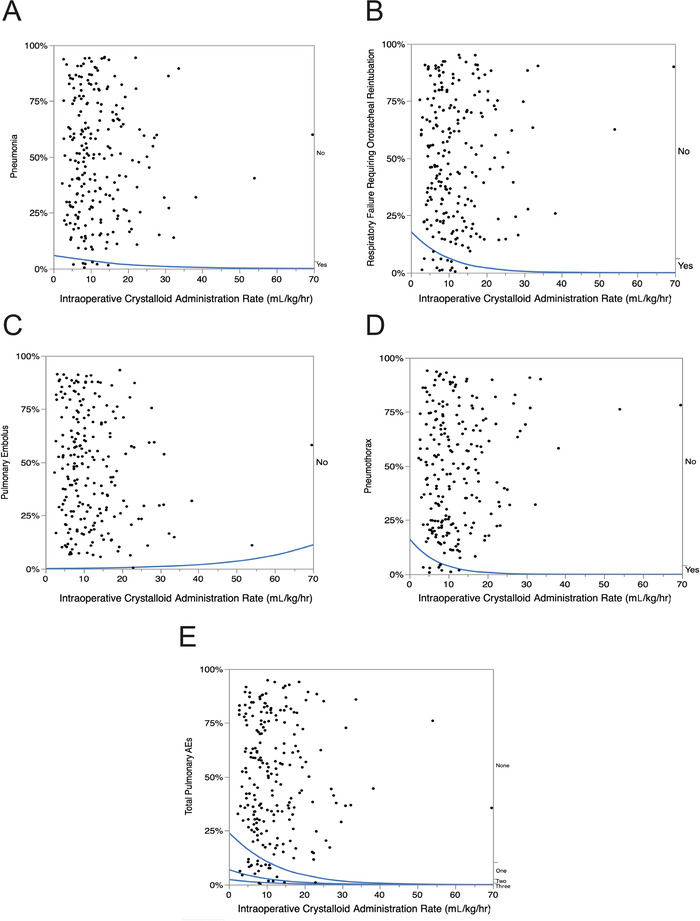
**Logistic fit of the role of intraoperative crystalloid administration (mL/kg/hr) on the incidence of common postoperative pulmonary adverse events (AEs) following thoracic surgery in 222 consecutive patients. The blue lines are the percent incidence fit of the dose-response relationship to pulmonary AEs. *P* values <0.01 are statistically significant**. **(A) Pneumonia: chi-square=0.9, *P*=0.3412; (B) Respiratory failure requiring orotracheal reintubation: chi-square=4.8, *P*=0.0281; (C) Pulmonary embolus: chi-square=0.85, *P*=0.3559; (D) Pneumothorax: chi-square=4.9, *P*=0.0276; (E) Total pulmonary AEs: chi-square=5.9, *P*=0.0155.**

Overall, the incidence of postoperative pulmonary AEs was 14.4% (CI 10.4%-19.6%). Dose-response graphs for the postoperative incidences of pneumonia, respiratory failure requiring orotracheal reintubation, pulmonary embolus, and pneumothorax are shown in Figures 4A to 4D. When the total number of postoperative pulmonary AEs (pneumonia [7 patients], respiratory failure requiring orotracheal reintubation [15 patients], pulmonary embolus [1 patient], and pneumothorax [9 patients]) was plotted against the rate of intraoperative crystalloid administration (Figure 4E), all AEs occurred at lower rates of intraoperative crystalloid administration.

#### Renal Adverse Events

**Figure 5.**
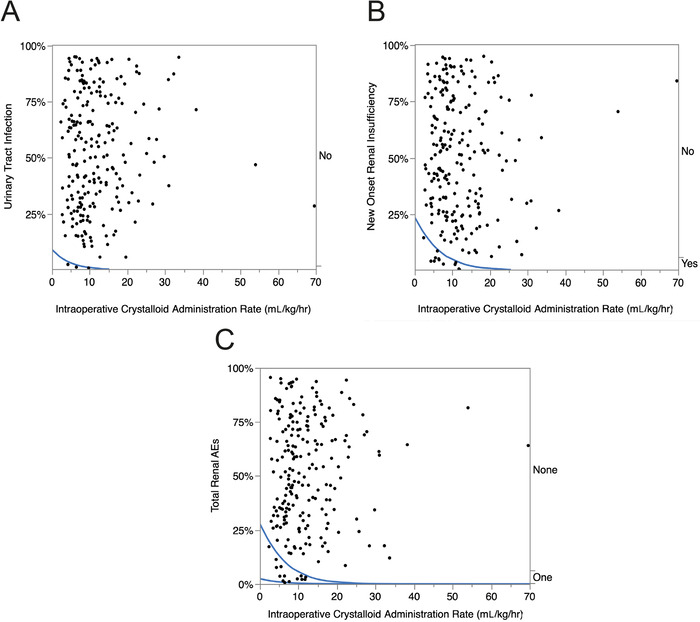
**Logistic fit of the role of intraoperative crystalloid administration (mL/kg/hr) on the incidence of common postoperative renal adverse events (AEs) following thoracic surgery in 222 consecutive patients. The blue lines are the percent incidence fit of the dose-response relationship to renal AEs. *P* values <0.01 are statistically significant**. **(A) Urinary tract infection: chi-square=2.5, *P*=0.1118; (B) New onset renal insufficiency: chi-square=7.5, *P*=0.0063; (C) Total renal AEs: chi-square=9.1, *P*=0.0025.**

Overall, the incidence of post-operative renal AEs was 6.8% (CI 4.1%-10.8%). Dose-response graphs for the postoperative incidences of urinary tract infection and new onset renal insufficiency are shown in Figures 5A and 5B. When the total number of postoperative renal AEs (urinary tract infection [3 patients] and new onset renal insufficiency [12 patients]) was plotted against the rate of intraoperative crystalloid administration (Figure 5C), all AEs occurred at lower rates of intraoperative crystalloid administration.

#### Other Adverse Events

**Figure 6.**
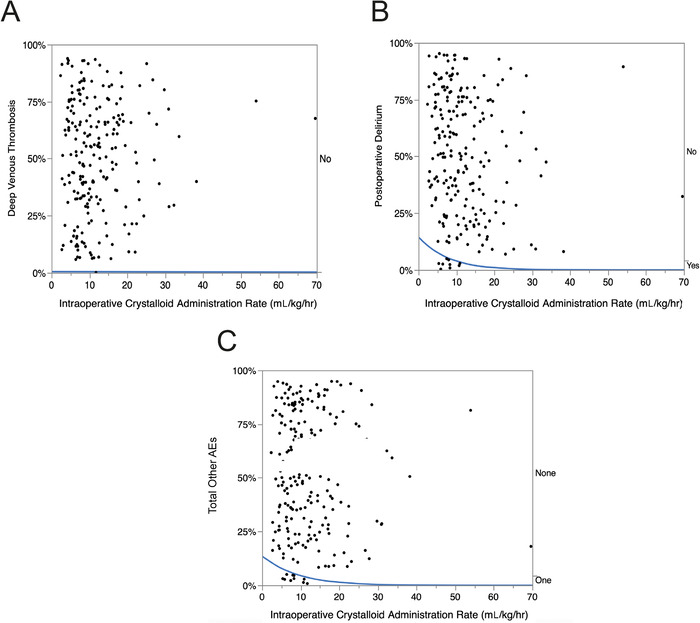
**Logistic fit of the role of intraoperative crystalloid administration (mL/kg/hr) on the incidence of common postoperative other adverse events (AEs) following thoracic surgery in 222 consecutive patients. The blue lines are the percent incidence fit of the dose-response relationship to other AEs. *P* values <0.01 are statistically significant**. **(A) Deep venous thrombosis: chi-square=0.002, *P*=0.9647; (B) Postoperative delirium: chi-square=4.1, *P*=0.0424; (C) Total other AEs: chi-square=3.6, *P*=0.0580.**

Overall, the incidence of postoperative other AEs was 4.5% (CI 2.5%-8.1%). Dose-response graphs for the postoperative incidences of deep venous thrombosis and postoperative delirium are shown in Figures 6A and 6B. When the total number of other postoperative AEs (deep venous thrombosis [1 patient] and postoperative delirium [9 patients]) was plotted against the rate of intraoperative crystalloid administration (Figure 6C), all AEs occurred at lower rates of intraoperative crystalloid administration.

#### Long-Term Adverse Events

**Figure 7.**
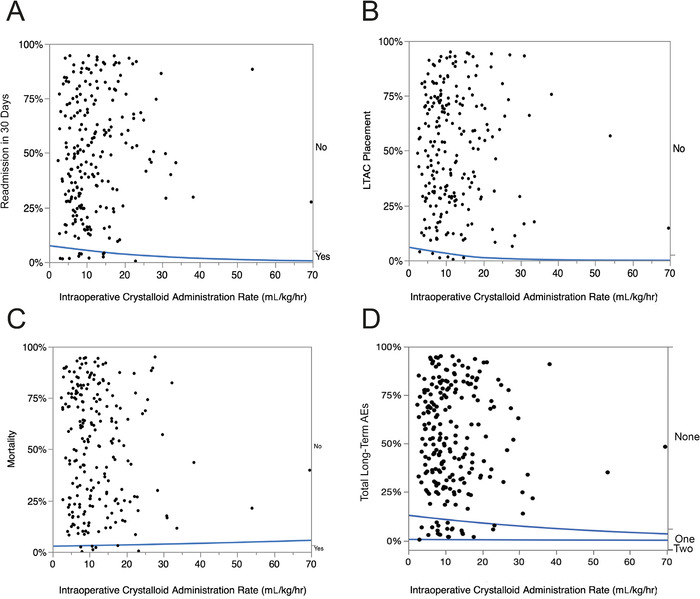
**Logistic fit of the role of intraoperative crystalloid administration (mL/kg/hr) on the incidence of long-term adverse events (AEs) following thoracic surgery in 222 consecutive patients. The blue lines are the percent incidence fit of the dose-response relationship to long-term AEs. *P* values <0.01 are statistically significant**. **(A) Readmission in 30 days: chi-square=0.7, *P*=0.4104; (B) Long-term acute care (LTAC) placement: chi-square=1.2, *P*=0.2822; (C) Mortality: chi-square=0.1, *P*=0.8107; (D) Total long-term AEs: chi-square=0.5, *P*=0.4980.**

Overall, the incidence of postoperative long-term AEs was 10.3% (CI 6.7%-14.8%). Dose-response graphs for the postoperative incidences of hospital readmission in 30 days, LTAC placement, and mortality are shown in Figures 7A to 7C. When the total number of long-term AEs (hospital readmission in 30 days [11 patients], LTAC placement [6 patients], and mortality [7 patients]) was plotted against the rate of intraoperative crystalloid administration (Figure 7D), these AEs occurred at lower rates of intraoperative crystalloid administration.

### Multivariable Analysis

A multivariable analysis of the predictors from Table 1, length of surgical operation, and rate of intraoperative crystalloid administration underwent stepwise screening, with only history of COPD, preoperative serum albumin, and rate of intraoperative crystalloid administration emerging as statistically associated with phLOS (Table 4). Reexamination of the analysis with the instrumental variable (Louisiana) showed no clinically important changes in parameter estimates, standard errors, *T* ratios, or VIF (Table 4).

**Table 4.** Multivariable Analysis of the Association of (A) Demographic Variables and Intraoperative Fluid Administration and (B) With the Instrumental Variable on Hospital Length of Stay in 222 Consecutive Patients Undergoing Thoracic Surgery

	Analysis A					
Parameter	Estimate	95% CI	SE	*T* Ratio	*P* Value	VIF
Intercept	21.1	16.1 to 26.1	2.5	8.3	<0.0001	
Chronic obstructive pulmonary disease	2.4	1.5 to 3.4	0.5	5.0	<0.0001	1.02
Preoperative serum albumin	–3.2	–4.6 to –1.8	0.7	–4.6	<0.0001	1.00
Intraoperative crystalloid administration rate	–0.17	–0.3 to –0.1	0.05	–3.4	0.0009	1.02

	**Analysis B**					
**Parameter**	**Estimate**	**95% CI**	**SE**	***T* Ratio**	***P* Value**	**VIF**

Intercept	21.5	16.5 to 26.5	2.5	8.5	<0.0001	
Chronic obstructive pulmonary disease	2.6	1.6 to 3.6	0.5	5.2	<0.0001	1.04
Preoperative serum albumin	–3.0	–4.4 to –1.7	0.7	–4.3	<0.0001	1.03
Intraoperative crystalloid administration rate	–0.16	–0.3 to –0.06	0.05	–3.3	0.0013	1.02
Regional instrumental variable [Louisiana]	–1.2	–2.6 to 0.1	0.7	–1.8	0.0763	1.04

VIF, variance inflation factor.

### Comparative Studies of Intraoperative Volume Administration

When phLOS was expressed as mean and SD, comparisons to other fluid administration studies in lung resection surgeries could be examined (Table 5).^35-38^ In a study of 879 patients, Licker, de Perrot, and colleagues^35^ reported a mean phLOS of 10.3 days, SD 2.4 days in a subset of patients (842/879, 95.8%) without acute lung injury. In this subset of patients, the mean intraoperative infusion rate was 7.2 mL/kg/hr, SD 4.2 mL/kg/hr. We observed a mean phLOS of 6.7 days, SD 6.5 days with an intraoperative fluid infusion rate of 12.3 mL/kg/hr, SD 8.3 mL/kg/hr (Table 5).

**Table 5.** Comparisons of Intraoperative Fluid Administration (IFA) to Postoperative Hospital Length of Stay (phLOS) in Patients Following Thoracic Surgery

	Comparable Studies		Present Study			
Study	IFA, mL/kg/hr, Mean, SD	phLOS, Days, Mean, SD	IFA mL/kg/hr, Mean, SD	phLOS, Days Mean, SD	phLOS Z Test Statistic	*P* Value
Licker et al, 2003,^35^ n=842/879	7.2, 4.2^a^	10.3, 2.4	12.3, 8.3^a^	6.7, 6.5	–22.0	<0.0001
Licker et al, 2009,^36^ n=558	3.5, 1.6^a^	11.8, 4.1	12.3, 8.3^a^	6.7, 6.5	–18.6	<0.0001
Licker et al, 2009,^36^ n=533	3.6, 1.4^a^	14.5, 3.3			–35.3	<0.0001
Matot et al, 2013,^37^ n=51	2.0^b^	5.8, 2.7	12.1, 8.2^b^	6.1, 6.2	1.5	0.1455
Matot et al, 2013,^37^ n=51	8.0^b^	5.5, 3.4			3.9	<0.0001
Arslantas et al, 2015,^38^ n=139	5.7, 3.2^a^	8.5, 4.8	12.3, 8.3^a^	6.7, 6.5	–5.7	<0.0001

^a^Patients received intraoperative crystalloids, colloids, and packed red blood cells.

^b^Patients only received intraoperative crystalloids.

Note: *P* values <0.01 are statistically significant.

Licker, Diaper, and colleagues^36^ repeated their examination of the association of mean intraoperative fluid administration rates in 2 cohorts—3.5 mL/kg/hr, SD 1.6 mL/kg/hr and 3.6 mL/kg/hr, SD 1.4 mL/kg/hr—with reported mean phLOS of 11.8 days, SD 4.1 days and 14.5 days, SD 3.3 days, respectively (Table 5). Again, we observed a mean phLOS of 6.7 days, SD 6.5 days with an intraoperative fluid infusion rate of 12.3 mL/kg/hr, SD 8.3 mL/kg/hr.

Matot et al^37^ examined the role of intraoperative fluid management in 102 patients undergoing video-assisted thoracoscopic surgery. Patients were randomized to receive either a 2 mL/kg/hr or 8 mL/kg/hr crystalloid administration rate during surgery (Table 5). Duration of phLOS was not different between their 2 groups: mean phLOS of 5.8 days, SD 2.7 days and 5.5 days, SD 3.4 days, respectively. A key finding in this study was no intraoperative administration of blood products or colloids.^37^ We had a subset of patients who only received intraoperative crystalloids, and their mean phLOS decreased to 6.1 days, SD 6.2 days (Table 5).

Arslantas et al^38^ examined the role of intraoperative fluid (crystalloids, colloids, and blood products) administration in 139 patients undergoing lung resection surgery (Table 5). Mean postoperative phLOS was 8.5 days, SD 4.8 days with a mean intraoperative fluid infusion rate of 5.7 mL/kg/hr, SD 3.2 mL/kg/hr.^38^ In our patients who received crystalloids, colloids, and blood products, the mean phLOS was lower at 6.7 days, SD 6.5 days, and the mean intraoperative fluid administration rate was higher at 12.3 mL/kg/hr, SD 8.3 mL/kg/hr (Table 5).

### Analyses to Support Causality

We conducted and analyzed additional tests to provide support for causality in the role of intraoperative crystalloid administration on duration of phLOS.^21,23,33^ Graphing the association of intraoperative crystalloid administration to duration of phLOS as a dose-response relationship or expressed as mL/kg/hr tertile groups clearly demonstrated improved clinical reductions in duration and variance of phLOS. This relationship supports biological plausibility and has been shown in other fluid administration studies when expressed as dose-response curves.^10,12-14^ Although retrospective studies risk effect-cause discovery, there was at least a 2-day spatial difference between crystalloid administration rate and hospital discharge in this study.^21,23,33^ This spatial difference suggests no effect-cause. The role of confounding was tested with an instrumental variable to measure the effects of unmeasured confounders. VIF calculations assessed for multicollinearity of the predictor variables.^24^ Omitted variable bias was minimized as 24 predictors that had been previously reported in thoracic surgery fluid administration studies were included for statistical analysis in this study.^5-9^ Exclusion bias was minimized through the study of consecutive patients undergoing thoracic surgeries. Cognitive bias was minimized through structured analysis of previously reported predictors with multivariable analysis, as well as the predictors observed to be significant with phLOS, the outcome of interest.^21,23^ However, referral bias is a common limitation observed in academic tertiary centers, and these data should only be interpreted in this type of clinical setting.^39^ Finally, false discovery rates were utilized to minimize the declarations of significance attributable to chance alone.^16,17^

## DISCUSSION

In this study of 222 patients undergoing lung resection, the rate of intraoperative crystalloid administration was causal to duration of phLOS. An additional key finding was the observation of higher variances in phLOS occurring with lower rates of intraoperative crystalloid administration.

The administration of colloids and of pRBCs increased phLOS, with pRBCs showing indirect evidence of no relation to length of surgery, EBL, or unplanned SICU admission. These results suggest that the administration of IV foreign proteins into the systemic circulation during thoracic surgery generates a systemic inflammatory response that delays hospital discharge.^40-43^

Analysis of previously reported postoperative AEs demonstrated clustering of these events at lower rates of intraoperative crystalloid administration. The observation that the associations of the independent predictor to outcomes are also in the shape of J-curves confirms reports in the medical literature that risk factor associations rarely exist on absolute incidence scales.^26,44-46^ These associations clearly suggest that intraoperative restriction of crystalloids does not favor successful outcomes in this patient population. Moreover, study of these dose-response relationships allows investigators to optimize future infusion rates to lessen or eliminate AEs in patients, an analysis that cannot be determined when data are summated.

The multivariable analysis suggested that of the 24 previously reported predictors of phLOS, only 3 predictors were associated with phLOS duration. Two of the 3 predictors in this patient population probably could not be modified—history of COPD and preoperative serum albumin levels—whereas the rate of intraoperative crystalloid administration is a readily adjustable predictor affecting outcomes in this patient population.

### Comparisons of Summation Studies

The fluid management studies of Licker, de Perrot, and colleagues^35^; Licker, Diaper, and colleagues^36^; and Arslantas et al^38^ all had lower rates of intraoperative fluid administration but higher mean phLOS when compared to the results of our study. These results suggest that a more liberal administration of crystalloids during lung resection surgery does not increase phLOS but rather allows earlier discharge from the hospital. The worsening of postoperative outcomes is attributable to an inflammatory response from the IV infusion of foreign proteins into the systemic circulation rather than the measurement of crystalloids used to dilute pRBCs when intraoperatively administered.^40-43^

### Causality

The tests used to support the likelihood of causality followed structured guidelines.^21,23,33^ When current observations confirm results observed in prior studies, the new associations are more likely to represent a true clinical effect.^21,23,33^ The strength of effect of intraoperative crystalloid administration to duration of phLOS underwent recursive partitioning to show a progressive decrease in phLOS and in phLOS variance across the calculated tertile ranges that were statistically significant compared with the higher tertile groups and with multivariable analysis to reduce the incidence of false discovery rates.^16,17^ Instrumental variable analysis revealed no significant change in the association of intraoperative crystalloid administration to phLOS. These results suggest that unmeasured confounders did not play a role and further support causality.^18-21,23^

### Strengths and Limitations

This investigation is a single-center study with referral bias, and the approaches and methods need to be investigated at other centers. Nevertheless, this study was a consecutive examination that underwent analyses with internal model validations to reduce overfitting in a group of patients undergoing common surgical procedures.

One limitation of retrospective studies is the use of incomplete records for data extraction that requires imputation strategies to use those records.^47^ However, as observed in this study, electronic medical records can allow for near 100% data harvest (0.04% missing data in this study). Another limitation of all studies is potential bias due to confounding. However, in this study, we measured 24 previously reported predictors^5-9^ and the predictors statistically associated with the primary outcome of interest (phLOS) via robust statistical analysis, including assessments for multicollinearity.^24^ In this analysis, VIF values were low (∼1), suggesting no multicollinearity between the independent variables. Additionally, instrumental variable analysis was employed to adjust for unknown confounders. For this study, the geographic region in which the patients reside was chosen to represent unmeasured confounders in this population that may or may not have an association with the outcome of interest.^18-21,23^ Another limitation is that this investigation is retrospective and was not conducted as a randomized controlled clinical trial. Although a randomized controlled trial may account for the effects of known and unknown confounders,^21,23^ not all patients will participate, and with the additional exclusion criteria inherent in this type of trial, such a study may not represent the population of interest.^48-50^ Moreover, current clinical findings and additional hospital costs would need to be considered for this type of study design.^48-50^ Instrumental variable analysis is frequently used in retrospective studies to mimic randomized controlled clinical trials, and the use of this statistical technique in this study strengthened the analysis in this consecutive patient population.^18-21,23^ Observations research is the preferred design for discovery of AEs.^51^ An additional strength of this study is the use of statistical techniques to lower the incidence of false discovery rates that guard against incorrect declarations of statistical significance leading to potential changes in clinical care that are attributable to chance alone.^16,17^ Another strength of this study is the use of recursive partitioning that grouped patients into different levels of risk, rather than through arbitrary divisions.^27-32^ Statistical tests that set *P* values for significance at <0.05 have a 30% chance of false-positive results with the risk of misdirected care.^16^ The false discovery rate is the complement of the positive predictive value with the probability of the statistically significant results supporting causality. In the present study, statistical significance and false discovery rates were set lower, resulting in a positive predictive value ranging from 93.3% to 98.2% for the intraoperative predictor to duration of phLOS.^16^ Finally, another strength of this study is the use of 7 recognized cause-and-effect concepts^21,23,33^ and instrumental variable analysis,^18-21^ supporting causality of the intraoperative predictor to phLOS.

## CONCLUSION

In this retrospective study of 222 consecutive patients undergoing thoracic surgery, rates of intraoperative crystalloid administration were causal in duration of phLOS, associated with the variance of phLOS, and associated with the incidences of commonly observed AEs. Causality of the intraoperative predictor to phLOS was supported with cause-and-effect and instrumental variable analyses and the use of false discovery rates. The dose-response graphs provide additional clinical information not clearly observed when associations are expressed as summations. Finally, these data suggest that a liberal and not a restrictive crystalloid administration strategy during thoracic surgery is firmly supported.

## Notes

*This article meets the Accreditation Council for Graduate Medical Education and the American Board of Medical Specialties Maintenance of Certification competencies for Patient Care, Medical Knowledge, and Practice-Based Learning and Improvement.*
